# Accurate clinical detection of exon copy number variants in a targeted NGS panel using DECoN

**DOI:** 10.12688/wellcomeopenres.10069.1

**Published:** 2016-11-25

**Authors:** Anna Fowler, Shazia Mahamdallie, Elise Ruark, Sheila Seal, Emma Ramsay, Matthew Clarke, Imran Uddin, Harriet Wylie, Ann Strydom, Gerton Lunter, Nazneen Rahman

**Affiliations:** 1 Wellcome Trust Centre for Human Genetics, University of Oxford, Oxford, UK; 2 Division of Genetics and Epidemiology, Institute of Cancer Research, London, UK; 3 TGLclinical, Institute of Cancer Research, London, UK; 4 Cancer Genetics Unit, The Royal Marsden NHS Foundation Trust, Sutton, UK

**Keywords:** Exon CNV, mutation testing, NGS, CNV, BRCA1, BRCA2, genetic testing, clinical genomics

## Abstract

**Background:** Targeted next generation sequencing (NGS) panels are increasingly being used in clinical genomics to increase capacity, throughput and affordability of gene testing. Identifying whole exon deletions or duplications (termed exon copy number variants, ‘exon CNVs’) in exon-targeted NGS panels has proved challenging, particularly for single exon CNVs.

**Methods:** We developed a tool for the Detection of Exon Copy Number variants (DECoN), which is optimised for analysis of exon-targeted NGS panels in the clinical setting. We evaluated DECoN performance using 96 samples with independently validated exon CNV data. We performed simulations to evaluate DECoN detection performance of single exon CNVs and to evaluate performance using different coverage levels and sample numbers. Finally, we implemented DECoN in a clinical laboratory that tests *BRCA1* and *BRCA2* with the TruSight Cancer Panel (TSCP). We used DECoN to analyse 1,919 samples, validating exon CNV detections by multiplex ligation-dependent probe amplification (MLPA).

**Results:** In the evaluation set, DECoN achieved 100% sensitivity and 99% specificity for BRCA exon CNVs, including identification of 8 single exon CNVs. DECoN also identified 14/15 exon CNVs in 8 other genes. Simulations of all possible BRCA single exon CNVs gave a mean sensitivity of 98% for deletions and 95% for duplications. DECoN performance remained excellent with different levels of coverage and sample numbers; sensitivity and specificity was >98% with the typical NGS run parameters. In the clinical pipeline, DECoN automatically analyses pools of 48 samples at a time, taking 24 minutes per pool, on average. DECoN detected 24 BRCA exon CNVs, of which 23 were confirmed by MLPA, giving a false discovery rate of 4%. Specificity was 99.7%.

**Conclusions:** DECoN is a fast, accurate, exon CNV detection tool readily implementable in research and clinical NGS pipelines. It has high sensitivity and specificity and acceptable false discovery rate. DECoN is freely available at www.icr.ac.uk/decon.

## Introduction

Targeted next generation sequencing (NGS) panels are increasingly being used in clinical genomics to increase capacity, throughput and affordability of gene testing ^1– 3^. For NGS panels to be effective in the clinical setting, all variant classes need to be robustly detected. Base substitutions are accurately detected by most pipelines and detection of small insertions and deletions are improving ^4– 6^. However, accurate detection of deletions or duplications of whole exons, also known as exon copy number variants (exon CNVs), has proved problematic in targeted NGS data, particularly detection of single exon CNVs ^7, 8^. In large part this is because the breakpoints usually lie outside the region targeted by the panel, and therefore detection methods are typically based on changes in the number of reads covering each target, commonly referred to as read depth or coverage. However, coverage can vary for several reasons, such as differences in GC content or individual probe efficiencies, and careful normalisation of data is therefore required ^7, 8^. These challenges have led many research and clinical laboratories to either ignore exon CNVs or to use alternative detection methods ^9^. The latter can lead to substantial increases in the time and cost of tests.

The Mainstreaming Cancer Genetics (MCG) programme ( www.mcgprogramme.com) is working to increase access to cancer predisposition gene (CPG) testing ^10^. To implement this we have developed, in collaboration with Illumina, a NGS panel targeting cancer predisposition genes called the TruSight Cancer Panel (TSCP) http://www.illumina.com/products/trusight_cancer.html. Many CPGs are tumour suppressor genes that predispose to cancer when their functions are inactivated by loss-of-function mutations ^11^. Exon CNVs are an important class of such pathogenic mutations accounting for appreciable proportions of mutations in many genes, including *BRCA1* and *BRCA2* ^12^. Several methods have been used for their detection in the pre-NGS era, including multiplex ligation-dependent amplification (MLPA), multiplex amplifiable probe hybridisation (MAPH) and array-based comparative genome hybridisation (aCGH) ^9, 13, 14^. For NGS analysis to replace these tools a method for exon CNV detection with high sensitivity, specificity and acceptable false discovery rate is required. For use in clinical laboratories it is also essential that the required quality control checks are fully integrated into the pipeline, so that reporting of positive *and* of negative tests is robustly achievable.

Several tools to detect exon CNVs in NGS sequence data have been published, including ExomeDepth, XHMM, and CONTRA ^15– 17^. Generally, these were developed for the research setting and for whole exome rather than targeted exon panels. The tools typically use coverage data from a set of samples as input, but may use different approaches for calling variants. For example, ExomeDepth selects samples from the input set that are well correlated with the sample of interest, and then fits a Beta-binomial model to the sample of interest and the selected samples ^16^. By contrast, XHMM performs principal component analysis normalisation on the matrix of coverage values and fits a standard normal model to the results ^17^. CONTRA creates a baseline from the input set of samples and models the log ratio of the sample of interest and the baseline with a normal distribution ^15^.

Here we have modified ExomeDepth to develop a tool, Detection of Exon Copy Number (DECoN), which is easy to implement and integrate in clinical laboratory pipelines and can display results in an interactive GUI for user-friendly data visualisation. With extensive real and simulated data we show that DECoN has high sensitivity and specificity and can be used as the first-line exon CNV detection tool in exon-targeted NGS panel analysis.

## Methods

### Samples and consent

We included data from 2,016 samples, 96 samples in the evaluation set and 1,920 samples in the clinical implementation set. Data were generated on lymphocyte DNA extracted from peripheral blood or saliva. Samples in the evaluation set were from individuals recruited to our studies into discovery and characterisation of disease predisposition genes, which have been approved by the London Multicentre Research Ethics Committee (05/MRE02/17, MREC/01/2/044, MREC/01/2/18), or from TGLclinical laboratory, an ISO18519 accredited genetic testing laboratory. Written informed consent to participate in the research studies or to have clinical genetic testing performed (as appropriate) was obtained from all participants. Samples in the clinical implementation set were from the TGLclinical laboratory. The TGLclinical data reported here were analysed as part of the TGLclinical accreditation and validation processes.

### Evaluation set

The evaluation set included 96 samples, including 10 samples with exon CNVs in *BRCA1*, six with exon CNVs in *BRCA2*, and 15 samples with exon CNVs in one of eight other genes: *TP53, SDHB, MLH1, MSH2, NSD1, EZH2, WT1* and *FH* ( Table 1). The remaining 65 samples were negative for BRCA exon CNVs on MLPA, and it is assumed they are also negative for exon CNVs in the other eight genes because they either have small intragenic pathogenic mutations that fully account for their phenotype, and/or their phenotype is not consistent with an exon CNV in any of the genes.

**Table 1.** Exon CNVs in the evaluation set. A total of 31 known exon CNVs in the evaluation set that were previously identified by multiplex ligation-dependent probe amplification. CNV, copy number variant.

Gene	CNV
BRCA1	Exon 5-8 duplication
BRCA1	Exon 13 duplication
BRCA1	Exon 20 deletion
BRCA1	Exon 1-12 deletion
BRCA1	Exon 1-2 deletion
BRCA1	Exon 21-24 deletion
BRCA1	Exon 8-13 deletion
BRCA1	Exon 16 deletion
BRCA1	Exon 22 deletion
BRCA1	Exon 20-22 deletion
BRCA2	Exon 14-16 deletion
BRCA2	Exon 1-11 deletion
BRCA2	Exon 1-2 deletion
BRCA2	Exon 2 deletion
BRCA2	Exon 21 duplication
BRCA2	Exon 21-24 deletion
EZH2	Exon 1-20 deletion
FH	Exon 1-10 deletion
MLH1	Exon 16-19 deletion
MSH2	Exon 1-6 deletion
NSD1	Exon 1-2 deletion
NSD1	Exon 1-23 deletion
NSD1	Exon 1-5 deletion
NSD1	Exon 19-21 deletion
NSD1	Exon 9-13 deletion
NSD1	Exon 22 deletion
SDHB	Exon 1 deletion
SDHB	Exon 2-7 deletion
SDHB	Exon 3-8 deletion
TP53	Exon 1-11 deletion
WT1	Exon 1-10 deletion

### Clinical implementation set

The implementation set included 1,920 samples. One sample had suboptimal DNA quality and the data was excluded. Data from 1,919 samples were therefore included in the described analyses. Pre-existing negative BRCA MLPA data was available for 307 samples and was used to evaluate the specificity of DECoN. In the interest of patient confidentiality, individual-level sequencing data is not made available. We anticipate that users will have their own datasets against which to test DECoN. Please contact the authors if test data would be helpful. 

### TruSight Cancer Panel (TSCP) sequencing

TSCP data was generated on all samples. We prepared targeted DNA libraries from 50ng genomic DNA using TSCP and TruSight Rapid Capture kit (Illumina). We followed the manufacturer’s protocol with the exception of library enrichment pool complexity, which we performed in 48-plex. We sequenced a final 10pM pooled library on a HiSeq2500 platform set in Rapid-run mode following standard protocols: 96-plex pool per flow cell, TruSeq Rapid SBS Kit, 101 bp paired-end dual index run and onboard clustering. We used CASAVA v.1.8.2 to demultiplex and create FASTQs per sample from the raw base call (.bcl) files. The sequence reads, by sample, were then mapped to the human reference genome (hg19) using Stampy v.1.0.20 ^18^.

### Multiplex ligation-dependent probe amplification (MLPA)

MLPA was used to evaluate all calls detected by DECoN using the appropriate probe kits and protocols from MRC Holland, as previously described ^14^.

### Detection of Exon Copy Number (DECoN)

We performed a review of the available methods and elected to build a tool through modification and optimisation of ExomeDepth v.1.0.0 ^16^. This tool was chosen because of its performance and because it was open source and easy to modify. We have called the tool DECoN for Detection of Exon Copy Number.

To create DECoN, we introduced code and implementation optimisations of ExomeDepth. DECoN includes two important code modifications of ExomeDepth v.1.0.0. First it enables detection of variants affecting the first exon on a chromosome, as defined in the BED file, which was not included in previous versions. Second, the HMM transition probabilities were altered to depend upon the distance between exons, so that exons adjacent in the list of targeted regions are treated independently if they are located so far apart on the chromosome that the probability of a germline variant spanning both exons is negligible. These two modifications have also been incorporated into ExomeDepth from versions v.1.1.0 onwards.

DECoN also includes several features to enhance and broaden the usability of ExomeDepth. ExomeDepth is an R package and thus requires a knowledgeable R user to select, specify, and run the appropriate functions in the correct order to generate easily interpretable output. It also requires a number of dependencies, which themselves may have different versions depending on the user’s local R installation, potentially impacting the final output. DECoN optimises, standardises and automates the exon CNV calling and visualisation of ExomeDepth, implementing full version control using packrat ^19^. This careful approach ensures DECoN implementation is suitable for clinical laboratories, is consistent across user installations and is not affected by future changes of incorporated packages or their dependencies.

To provide a simple interface for users, DECoN requires only a set of BAM files, a BED file and a reference FASTA file. The user can supply a custom annotation file to suit their needs, for example to provide the relevant exon numbering for their genes of interest.

DECoN relies on a high level of correlation between samples, comparing the sample of interest only to those with which it is well correlated. The DECoN output reports the correlation between samples and the number of samples selected for comparison for every call. This is very useful as robust variant calling in the clinical setting requires information on potential suboptimal performance in order to report positive and negative results. DECoN also allows the user to set thresholds to flag samples and/or exons which may have suboptimal performance.

**Figure 1.**
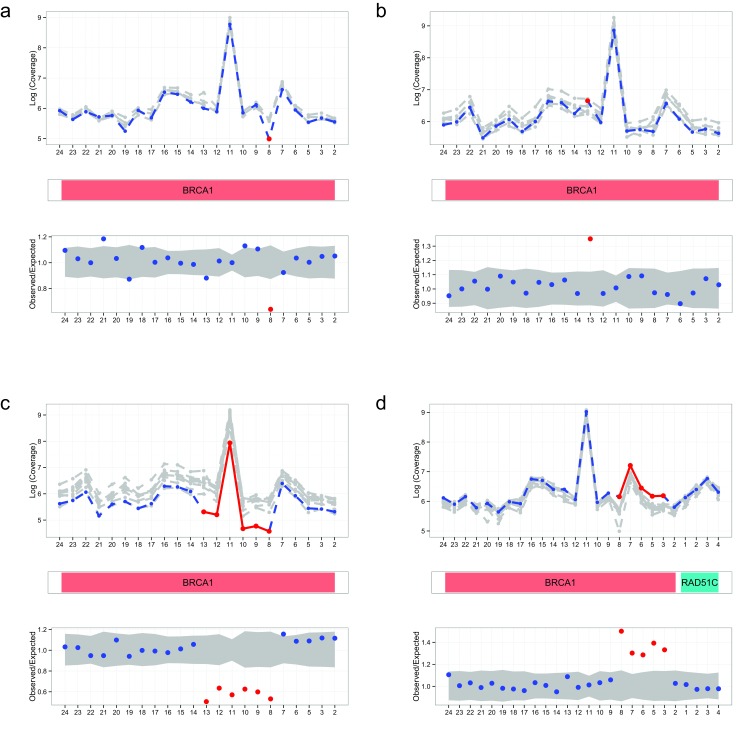
Automated visualisations from DECoN. In all panels, the top plot shows the log-normalised coverage of the sample of interest (blue) relative to reference samples (grey) and the bottom plot shows the ratio of observed to expected coverage with a 95% confidence interval in grey. The relevant gene(s) are shown between the plots in red. Deleted or duplicated exons called by DECoN are in red. ( **a**) A single exon deletion; ( **b**) A single exon duplication; ( **c**) A multi-exon deletion; ( **d**) A multi-exon duplication.

The DECoN output contains information on all exon CNVs called, their clinical annotations, and a list of regions and/or samples where calling may be suboptimal. An automatic visualisation of each result is generated as a PDF file; typical examples are shown in Figure 1. Furthermore, interactive visualisation of results is implemented using shiny: Web application framework for R (v 0.12.0) (available from shiny archive link on https://cran.r-project.org/web/packages/shiny/index.html) and can be launched in a modern browser such as Firefox, Chrome or Internet Explorer (v.10 or later) using a simple interface for Windows, Mac OSX and Linux operating systems. For example, the user can vary the plotting parameters interactively, enabling closer evaluation of supporting evidence and measures of confidence for specific calls as required.

The full DECoN documentation is given in Supplementary File 1. DECoN is publicly available from www.icr.ac.uk/decon and https://github.com/RahmanTeam/DECoN ^20^.

### Simulations

Detection of single exon CNVs is known to be particularly challenging ^7, 21, 22^. To better evaluate DECoN performance in single exon CNV detection we simulated single exon deletions and duplications in *BRCA1* and *BRCA2* in a single pool, using 48 samples from the evaluation set that were known to be negative for BRCA exon CNVs. This simulated data was based on real data, using the variation and fluctuations observed in the true coverage to model the simulated coverage. To simulate a duplication or deletion of a single exon, the observed coverage of that exon in a randomly selected sample was increased or decreased by 50%, respectively. This was repeated 1,000 times for each possible variant. Sensitivity was calculated as the percentage of the 1,000 repeats which were successfully detected.

We also performed simulations to evaluate the effect of varying the coverage and/or the number of samples in an enrichment pool. Simulated data was generated based on the evaluation set by first selecting an enrichment pool, then selecting samples from that enrichment pool for up or down sampling. When selecting 96 samples, all samples from the evaluation set were used. For any read, *r*, it was assumed to contribute N _*r*_ times to the simulated data set, where N _*r*_ was drawn from a Binomial ( *n*, *p*) distribution. This was assumed for all reads from the selected samples. The values of *n* and *p* were chosen to provide the correct level of up or down sampling and the closest approximation to a Poisson distribution, which the original coverage values are assumed to follow. Sensitivity and specificity were determined by assessing detection of variants known to be present in the original data. Simulated datasets which did not contain any samples with an exon CNV were excluded from the sensitivity calculations and simulated datasets entirely comprised of samples with exon CNVs were excluded from the specificity calculations.

## Results and discussion

### DECoN evaluation

To evaluate sensitivity and specificity, we applied DECoN to the evaluation set data. DECoN had a 100% sensitivity for BRCA exon CNVs (16/16). The specificity for BRCA exon CNVs was 99% as an exon 8-11 duplication in *BRCA1* was not confirmed by MLPA. Inspection of the DECoN visualisation showed this was to be expected, as the trace did not show the clear separation from the reference samples observed in true CNVs ( Supplementary File 2a). DECoN’s excellent performance was particularly striking as 33% of samples in one of the evaluation pools had BRCA exon CNVs (16/48), a highly unlikely scenario in clinical testing. This might have compromised detection, due to the higher chance of selecting a sample in the reference set that also has a CNV. It is thus very reassuring that DECoN performed so well in this extreme setting. Amongst the other genes, only an exon 22 deletion in *NSD1* was not detected, giving a sensitivity of 93% (14/15). Inspection of the DECoN visualisation showed this exon was below the reference samples, but the drop in coverage for this single exon was not sufficiently large for automatic detection using the default parameters ( Supplementary File 2b). Altering the transition probability would allow DECoN to flag this variant, but would also increase the false discovery rate. Users of DECoN can alter the parameters according to their needs and their required balance of the inevitable trade-off between sensitivity and false discovery. Of note, the *NSD1* exon 22 deletion was successfully automatically detected by DECoN on repeat testing ( Supplementary File 2c).

### Simulation analyses

**Figure 2.**
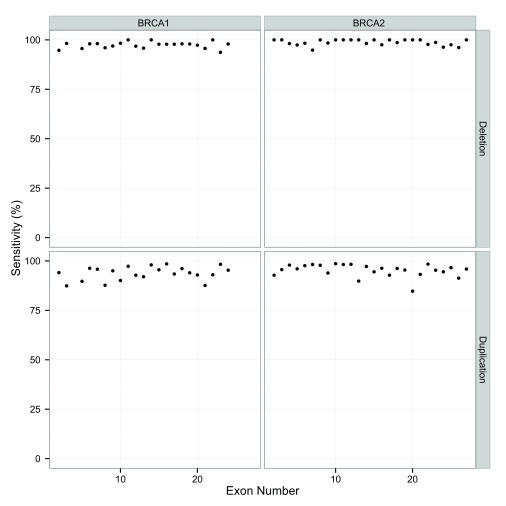
Sensitivity results for simulated single exon CNVs in *BRCA1* and *BRCA2.* Sensitivity is calculated as the percentage of correct detection of a given exon CNV out of 1,000 simulations. Exons are numbered within each gene according to the direction of the transcript.

To further explore the performance of DECoN in the detection of single exon CNVs we simulated a single exon deletion and duplication for each of the exons in *BRCA1* and *BRCA2* using the observed TSCP data from the evaluation set. The sensitivity for each exon deletion or duplication is shown in Figure 2. Sensitivity for single exon deletions was excellent, >94% in every exon with a mean of 98%. The sensitivity for single exon duplications was somewhat lower, with a mean of 95%, but these are known to be the most challenging exon CNV to detect.

In targeted sequencing, there are several experimental parameters that can impact exon CNV detection. For example, enrichment and/or sequencing can be performed in pools of different sample sizes. This will affect the coverage per sample and the number of samples available to use as reference samples. In turn this could affect DECoN performance. To evaluate this we extended our simulation framework to test *in silico* the effect of different combinations of sample size and coverage on DECoN’s sensitivity and specificity.

The 96 samples in the evaluation set were sequenced in a single HiSeq2500 run using Rapid-run mode and a single flow cell which outputs a maximum of 300 Million (M) read pairs per run, and thus have a maximum of 3.125M read pairs per sample. By comparison, a MiSeq platform using the reagent kit v2 can produce a maximum of 15M read pairs per run. The evaluation set was thus up-sampled or down-sampled to obtain varying numbers of reads per sample. DECoN was then run on each simulated set and the sensitivity and specificity were calculated using the known exon CNVs in the evaluation set.

The simulation results are shown in Table 2. If the sample size was reduced to six samples sequenced at 1.25M reads per sample, the sensitivity was compromised (87%), but if the six samples were sequenced with coverage ≥ 2.5M reads per sample, sensitivity of ≥ 95% was still achievable. Sequencing 12 samples at 1.25M reads per sample, compatible with a typical MiSeq run, produced good sensitivity (92%) and excellent specificity (99%). In general, sequencing higher sample sizes per pool increased sensitivity, but if at least 2.5M reads per sample are generated, the estimated sensitivity was ≥ 95% for all sample sizes evaluated ( Table 2).

**Table 2.** Results of simulations varying coverage and sample numbers. Simulations of DECoN exon CNV calling in the evaluation set, using different coverage and sample numbers, were performed. Average sensitivity and specificity values are shown, with the range in parentheses. Sensitivity and specificity achievable by an Illumina sequencing run generating 15M read pairs (typical of a MiSeq run) and 300M read pairs (typical of a HiSeq2500 Rapid run) are shown in green and blue, respectively.

		Number of samples per sequencing pool				
**Coverage per** **sample in million** **(M) read pairs**		6	12	24	48	96
1.25	Sensitivity	87% (0–100)	92% (40–100)	92% (60–100)	91% (81–100)	96% (94–100)
	Specificity	98% (56–100)	99% (89–100)	99% (96–100)	99% (98–100)	99% (99–100)
2.5	Sensitivity	95% (0–100)	98% (67–100)	99% (83–100)	100% (94–100)	100% (100–100)
	Specificity	97% (50–100)	99% (89–100)	99% (95–100)	99% (98–100)	99% (99–99)
3.125	Sensitivity	99% (67–100)	99% (75–100)	100% (100–100)	100% (100–100)	100% (100–100)
	Specificity	97% (50–100)	99% (94–100)	100% (97–100)	99% (99–100)	99% (99–99)
5	Sensitivity	96% (0–100)	99% (75–100)	100% (100–100)	100% (100–100)	100% (100–100)
	Specificity	97% (50–100)	99% (84–100)	99% (93–100)	99% (96–100)	99% (98–99)
7.5	Sensitivity	96% (0–100)	99% (75–100)	100% (89–100)	100% (94–100)	100% (100–100)
	Specificity	97% (56–100)	99% (84–100)	99% (92–100)	99% (96–100)	99% (98–99)

### DECoN clinical implementation

DECoN was incorporated into a clinical pipeline for BRCA analysis using TSCP and applied to 1,919 samples. DECoN was run for each of the 40 pools of 48 samples and took, on average, 24 minutes for each pool (range 19–29 minutes). In total, 23 exon CNVs were detected by DECoN and confirmed by MLPA ( Supplementary File 3). One exon CNV (a single exon duplication) detected by DECoN was not confirmed by MLPA, a false discovery rate of 4% (1/24). No exon CNV calls were made by DECoN amongst the 307 samples with pre-existing negative MLPA data, yielding an overall specificity of 99.7% (307/308) and a false positive rate of 0.3% (1/308).

Due to DECoN’s excellent performance and its fast, easy, user-friendly interface it is now the standard first-line exon CNV detection method in both our research and clinical TSCP testing pipelines. DECoN runs automatically after the small variant calling, adding <30 minutes to the analysis time for batches of 48 samples, and then both the small variants and exon CNVs are outputted for interpretation and management.

## Conclusions

We have developed, validated and implemented a fast, accurate, high-throughput, exon CNV detection tool, which we have called DECoN. DECoN is suitable for both research and clinical pipelines, but we have particularly focussed on optimising it for the needs of clinical laboratories. To facilitate evaluation and implementation of DECoN by other groups we have made the packaged version we use available at www.icr.ac.uk/decon, together with comprehensive documentation. The latter is also available as Supplementary File 1. DECoN is also available on GitHub to allow access and flexibility for the research user ( https://github.com/RahmanTeam/DECoN) ^20^. We believe DECoN can serve as a first-line method for exon CNV detection in targeted panels analysed to detect small intra-exon variants, obviating the need for a separate, parallel method for their detection. In turn this can increase the performance, speed and cost-efficiency of NGS testing.

## Abbreviations

CNV: Copy number variant

GUI: Graphical user interface

HMM: Hidden Markov model

MLPA: Multiplex ligation-dependent probe amplification

TSCP: Illumina TruSight Cancer panel

## Software and data availability

DECoN with supporting information available from: www.icr.ac.uk/decon

Latest source code: https://github.com/RahmanTeam/DECoN

Archived source code at time of publication: 10.5281/zenodo.167780 ^20^

Licence: MIT

## Supplementary material

**Supplementary File 1. DECoN documentation.** Full DECoN v.1.0.0 documentation.

**Supplementary File 2. DECoN GUI visualisations.** GUI visualisations of the two exon CNVs with discrepant DECoN (using default settings) and multiplex ligation-dependent probe amplification (MLPA) results. For each, the top plot shows the log-normalised coverage of the sample of interest (blue) relative to reference samples (grey) and the bottom plot shows the ratio of observed to expected coverage with a 95% confidence interval in grey. The relevant gene is shown between the plots in red. Exon CNVs called by DECoN are in red. ( **a**) DECoN false positive call of BRCA1 exon 8-11 duplication. ( **b**) DECoN false negative call of NSD1 exon 22 deletion in first test, but correct call of the deletion in the repeat test ( **c**).

**Supplementary File 3. Exon CNV detections in the clinical implementation set.** Exon CNVs in the clinical implementation set detected by DECoN and confirmed by multiplex ligation-dependent probe amplification.

## References

[1] Rehm HL: Disease-targeted sequencing: a cornerstone in the clinic. *Nat Rev Genet.* 2013;14(4):295–300. 10.1038/nrg3463 23478348 PMC3786217

[2] Castéra L Krieger S Rousselin A: Next-generation sequencing for the diagnosis of hereditary breast and ovarian cancer using genomic capture targeting multiple candidate genes. *Eur J Hum Genet.* 2014;22(11):1305–13. 10.1038/ejhg.2014.16 24549055 PMC4200427

[3] Pua CJ Bhalshankar J Miao K: Development of a Comprehensive Sequencing Assay for Inherited Cardiac Condition Genes. *J Cardiovasc Transl Res.* 2016;9(1):3–11. 10.1007/s12265-016-9673-5 26888179 PMC4767849

[4] Ruark E Münz M Clarke M: OpEx - a validated, automated pipeline optimised for clinical exome sequence analysis. *Sci Rep.* 2016;6: 31029. 10.1038/srep31029 27485037 PMC4971567

[5] Hasan MS Wu X Zhang L: Performance evaluation of indel calling tools using real short-read data. *Hum Genomics.* 2015;9(1):20. 10.1186/s40246-015-0042-2 26286629 PMC4545535

[6] Lelieveld SH Veltman JA Gilissen C: Novel bioinformatic developments for exome sequencing. *Hum Genet.* 2016;135(6):603–14. 10.1007/s00439-016-1658-6 27075447 PMC4883269

[7] de Ligt J Boone PM Pfundt R: Detection of clinically relevant copy number variants with whole-exome sequencing. *Hum Mutat.* 2013;34(10):1439–48. 10.1002/humu.22387 23893877

[8] Tan R Wang Y Kleinstein SE: An evaluation of copy number variation detection tools from whole-exome sequencing data. *Hum Mutat.* 2014;35(7):899–907. 10.1002/humu.22537 24599517

[9] Ceulemans S van der Ven K Del-Favero J: Targeted screening and validation of copy number variations. *Methods Mol Biol.* 2012;838:311–28. 10.1007/978-1-61779-507-7_15 22228019

[10] George A Riddell D Seal S: Implementing rapid, robust, cost-effective, patient-centred, routine genetic testing in ovarian cancer patients. *Sci Rep.* 2016;6: 29506. 10.1038/srep29506 27406733 PMC4942815

[11] Rahman N: Realizing the promise of cancer predisposition genes. *Nature.* 2014;505(7483):302–8. 10.1038/nature12981 24429628 PMC4975511

[12] Smith MJ Urquhart JE Harkness EF: The Contribution of Whole Gene Deletions and Large Rearrangements to the Mutation Spectrum in Inherited Tumor Predisposing Syndromes. *Hum Mutat.* 2016;37(3):250–6. 10.1002/humu.22938 26615784

[13] Kousoulidou L Sismani C Patsalis PC: Multiplex Amplifiable Probe Hybridization (MAPH) methodology as an alternative to comparative genomic hybridization (CGH). *Methods Mol Biol.* 2010;653:47–71. 10.1007/978-1-60761-759-4_4 20721737

[14] Eijk-Van Os PG Schouten JP: Multiplex Ligation-dependent Probe Amplification (MLPA®) for the detection of copy number variation in genomic sequences. *Methods Mol Biol.* 2011;688:97–126. 10.1007/978-1-60761-947-5_8 20938835

[15] Li J Lupat R Amarasinghe KC: CONTRA: copy number analysis for targeted resequencing. *Bioinformatics.* 2012;28(10):1307–13. 10.1093/bioinformatics/bts146 22474122 PMC3348560

[16] Plagnol V Curtis J Epstein M: A robust model for read count data in exome sequencing experiments and implications for copy number variant calling. *Bioinformatics.* 2012;28(21):2747–54. 10.1093/bioinformatics/bts526 22942019 PMC3476336

[17] Fromer M Moran JL Chambert K: Discovery and statistical genotyping of copy-number variation from whole-exome sequencing depth. *Am J Hum Genet.* 2012;91(4):597–607. 10.1016/j.ajhg.2012.08.005 23040492 PMC3484655

[18] Lunter G Goodson M: Stampy: a statistical algorithm for sensitive and fast mapping of Illumina sequence reads. *Genome Res.* 2011;21(6):936–9. 10.1101/gr.111120.110 20980556 PMC3106326

[19] Ushey K McPherson J Cheng J: A Dependency Management System for Projects and their R Package Dependencies.2016 Reference Source

[20] Fowler A Mahamdallie S Ruark E: DECoN (Detection of Exon Copy Number) [Data set]. *Zenodo.* 2016 Data Source 10.12688/wellcomeopenres.10069.1 PMC5409526 28459104

[21] Amarasinghe KC Li J Halgamuge SK: CoNVEX: copy number variation estimation in exome sequencing data using HMM. *BMC Bioinformatics.* 2013;14(Suppl 2):S2. 10.1186/1471-2105-14-s2-s2 23368785 PMC3549847

[22] Krumm N Sudmant PH Ko A: Copy number variation detection and genotyping from exome sequence data. *Genome Res.* 2012;22(8):1525–32. 10.1101/gr.138115.112 22585873 PMC3409265

