# Supplementary material for: Accurate clinical detection of exon copy number variants in a targeted NGS panel using DECoN
Source: Wellcome Open Res. 2016 Nov 25;1:20. [Version 1] doi: 10.12688/wellcomeopenres.10069.1 (PMC5409526; doi:10.12688/wellcomeopenres.10069.1)
Supplement: Supplementary file 1 [file wellcomeopenres-1-10849-s0000.tgz › 80c56c13-aa58-488a-83a6-e0f78f0af2a6.docx]

**DECoN v1.0.0**

**Documentation**

**1 Introduction ------------------- Page 2**

**2 Dependencies ------------------- Page 2**

**3 Installation ------------------- Page 2**

**4 Running DECoN ------------------ Page 3**

**5 Examples ----------------------- Page 10**

**6 References---------------------- Page 19**

**7 Contact ------------------------ Page 19**

**8 Appendix ----------------------- Page 20**


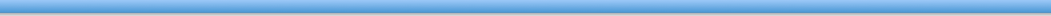


**1 INTRODUCTION**

DECoN (Detection of Exon Copy Number) is a sensitive and specific tool for detection of exon copy number variants (exon CNVs) in targeted sequencing data. DECoN provides quality checks and visualization to enhance utility for the clinical setting. DECoN is based on ExomeDepth^1^, optimised for use on targeted panels run on batches of samples. This tool has been developed through a collaboration between the Institute of Cancer Research, London and the Wellcome Trust Centre for Human Genetics, University of Oxford.

DECoN is implemented in R^2^ and has a strict version control using packrat^3^. It therefore will not be affected by future changes to any packages or their dependencies.

This detailed documentation describes all the features of DECoN. After first describing its user interface and user defined input thresholds, an example is presented in the second part of this documentation.


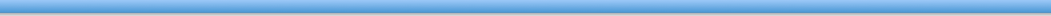


**2 DEPENDENCIES**

DECoN is implemented in R and requires

- R version 3.1.2.
- The capacity to build packages from source. See Section 3.1 for detailed instructions.
- A modern internet browser such as Firefox, Chrome, or IE v.10 or later.
- An internet connection for installation.


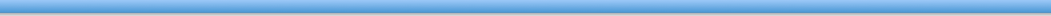


**3 INSTALLATION**

DECoN is available for Mac OS X, Linux, and Windows and can be downloaded from www.icr.ac.uk/DECoN. Installation of DECoN requires the following steps:

- The capacity to build R packages from source (Section 3.1)
- Installation (Section 3.2)

**3.1 Building packages from source**

The capacity to build R packages from source is required. Instructions for building packages from source in either a Mac/Linux environment or a Windows environment are provided below.

**3.1.1 Mac/Linux**

Building packages from source in a Mac or Linux environment requires gcc and gfortran compilers. The Mac gfortran compiler is available from <http://cran.r-project.org/bin/macosx/tools/>. gcc is available as part of xtools, the Mac command line tools.

**3.1.2 Windows**

Building packages from source in a Windows environment can be accomplished with Rtools. Detailed instructions for downloading and installing Rtools can be found in Section 8.

**3.2 Installation**

Installation of DECoN requires the following steps:

- Unpack the compressed file to a new local directory
- Run the setup script:
  - In a Mac/Linux environment – from the directory containing the DECoN scripts, run setup.sh from the command line.
  - In a Windows environment – run the setup.bat file. The file path to R version 3.1.2 (e.g. C:\Program Files\R\R-3.1.2\bin) must be in the PATH variable.

DECoN implements strict version control over all packages and dependencies used by changing the local default R settings. Any R session launched from the same directory as the DECoN scripts will have these settings, therefore it is recommended to unpack the compressed file to a new directory containing only DECoN.

The setup script downloads and installs all required packages and dependencies, automatically creating a log file. In a Mac/Linux environment, the log file is called setup.log. In a Windows environment, the log file is called setup.Rout and is located in the scripts folder.


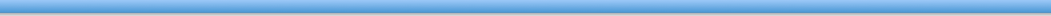


**4 RUNNING DECoN**

Once DECoN has been downloaded with dependencies correctly installed (see above), running DECoN requires four sequential steps:

1. Reading the BAM files to generate coverage metrics (Section 4.1)
2. Running quality checks (Section 4.2)
3. Calling exon CNVs(Section 4.3)
4. Visualizing the calls (Section 4.4)

A summary .RData file is created after steps 1 and 3 which is required for the following steps. This approach allows the user to perform individual steps without having to re-run preceding steps. For instance, a user can make calls with a different set of parameters or change quality thresholds for failing samples without having to re-process the BAM files.

In Mac/Linux, each step is launched via the command line. In Windows, there are executable files for each step which are clicked to launch. The user is then prompted to specify the required inputs. Detailed examples for a Mac/Linux and a Windows environment are provided in Sections 7.1 and 7.2, respectively.

**4.1 Reading BAM files to generate coverage metrics**

DECoN uses a list of BAM files and a BED file of exons to calculate a coverage metric called the fragment per kilobase and million base pairs (FPKM) for each exon specified in the BED file in each sample’s BAM file. DECoN uses this metric to call exon CNVs. FPKM is calculated according to the below formula:

FPKM = C/(N*L)

where C is the number of read pairs mapping to the exon, N is the total number of mapped read pairs divided by one million, and L is the length of the exon in kilobases.

For example, consider a sample with a total of 20 million mapped read pairs of which 200 map to an exon which is 100 bases long:

FPKM = 200/(20*0.1)

Thus FPKM for this exon in this sample is 100.

**4.1.1 Inputs**

There are four required inputs:

- BAM files – these can either be specified in a text file or a path to a directory containing all the BAM files can be supplied. The text file must contain a list of the BAM files to be read in, with each file name on a separate line and each file name ending in .bam. If a path is supplied, all BAM files in the directory will be read. DECoN expects each BAM file to have a .bai file in the same location as the .bam file, with a .bai extension instead of .bam as the file name, e.g. *directory/sample.bam* and *directory/sample.bai*.
- BED file – the targeted 1-based BED file to be used for analysis. This file must be sorted in chromosome order. This file does not have a header and must have four tab-separated columns corresponding to:
  - Chromosome
  - Start position
  - End position
  - Gene
- FASTA file – the reference genome FASTA file to be used with the data.
- Output prefix – the prefix for the summary output .RData file. If none is supplied, the default value is DECoN.

Examples of the input files are given in Section 5.1.

**4.1.2 Running ReadInBams**

In Mac/Linux, run the following command from the directory containing the DECoN scripts:

**Rscript ReadInBams.R --bams *bams.file* --bed *bed.file* --fasta *fasta.file* --out *output.prefix***

In Windows, click the *ReadInBams.bat* executable. You will be prompted to enter inputs.

**4.1.3 Output**

ReadInBams outputs a summary .RData file prefixed with the output prefix specified in the input which contains sample coverage values and sample names taken from the BAM files.

**4.2 Running quality checks**

The summary .RData file outputted in Section 4.1.3 can be used to flag any samples or exons where exon CNV calling may be suboptimal. Both exons and samples are evaluated based on their median coverage level. When coverage is low, accuracy of detection will be compromised and caution should be exercised when interpreting results. Samples are also evaluated based on their correlation with other samples. Samples which do not have a high correlation with other samples in the set are likely to have suboptimal detection across the entire target. Recommended default thresholds underpinning this quality flag are given below.

**4.2.1 Inputs**

There is one required file input and a number of threshold inputs which can be set by the user:

- Summary RData file (required) – A summary RData file containing the FPKM for each exon of an analyzed BED file, created in Section 4.1.3.
- Minimum correlation threshold – the minimum correlation between a test sample and any other sample for the test sample to be considered well-correlated. The default value is 0.98.
- Minimum coverage threshold – the minimum median coverage for any sample (measured across all exons in the target) or exon (measured across all samples) to be considered well-covered. The default value is 100.
- Exon numbering (optional) – a file containing exon numberings with custom annotation for at least one value in the analyzed BED file. This is a tab-separated file with four columns labelled with headings:
  - **Chromosome**
  - **Start** – start position from the analyzed BED file
  - **End** – end position from the analyzed BED file
  - **Custom.Exon** – custom exon name
- Custom reporting – Boolean value indicating whether an output file containing samples or exons which affect genes with custom annotation should be generated. The default value is FALSE.
- Output prefix – the prefix for the output files. If none is supplied, the default value is DECoN.

**4.2.2 Running IdentifyFailures**

In Mac/Linux, run the following command from the directory containing the DECoN scripts:

**Rscript IdentifyFailures.R --Rdata *summary.file* --mincorr *.98* --mincov *100*  --exons *customNumbers.file* --custom *FALSE* --out *output.prefix***

In Windows, click the *IdentifyFailures.bat* executable. You will be prompted to enter inputs.

**4.2.3 Outputs**

If all samples and exons are above the user defined thresholds, no output is created. If any suboptimal samples and/or exons are identified, a tab-separated text file ending in *_Failures.txt* is created with six columns:

- **Sample** – the name of the sample. If an exon is suboptimal this column has value “All”.
- **Exon** – the number of the suboptimal exon, in the order of the analyzed BED file. If the sample has suboptimal correlation or suboptimal median coverage, this column has value “All”.
- **Type** – the type of failure, either “Whole sample” if the sample is below the correlation or coverage threshold or “Whole exon” if the exon is below the coverage threshold.
- **Gene** – the name of the gene, from the Gene column of the analyzed BED file.
- **Custom.numbering** – the number of the suboptimal exon from the Custom.Exon column of the custom numbering file.
- **Info** – the underlying metric information.

If the custom option is TRUE and exons are identified which affect a gene with custom annotation, an additional file ending in *_custom_Failures.txt* is created. This is the same format as the *_Failures.txt* file and contains the subset of information pertaining to the relevant genes.

**4.3 Calling exon CNVs**

This step calls exon CNVs in each sample by selecting reference samples from all other samples contained in the input summary .RData file. The correlation between samples and the number of samples used as a reference are thus calculated and outputted to aid interpretation of call quality.

The HMM transition probabilities are altered from ExomeDepth v1.0.0. to depend upon the distance between exons, so that exons adjacent in the list of targeted regions are treated independently if they are located so far apart on the chromosome that the probability of a germline variant spanning both exons is negligible, specifically:

- The probability of transitioning into a CNV state (from normal to deletion or from normal to duplication) is given by a constant transition probability specified by the user (set as default to .01).
- The probability of transitioning to a normal state from a CNV state (from deletion to normal or from duplication to normal) is given by a baseline probability scaled by the distance between exons. If the distance between these exons is 0, then this scaling factor is simply 1, but as the distance increases, the scaling factor tends to 0. This is given by

$$\exp\left( -\frac{l}{E} \right)*1/t$$

where $l$ is the distance from the previous exon; E is the expected CNV length in basepairs; and t is the baseline probability of returning to a normal state from a deletion/duplication. These values are set as E=50000 and t=.5.

**4.3.1 Inputs**

There is one required file input and a number of parameter inputs which can be set by the user:

- Summary RData file (required) – A summary RData file containing the FPKM for each exon of an analyzed BED file, created in Section 4.1.3.
- Transition probability – the transition probability between normal copy number state and either deletion or duplication state in the hidden Markov model. The default value is set to 0.01, a high threshold value to increase sensitivity.
- Exon numbering (optional) – a file containing exon numberings with custom annotation for at least one value in the analyzed BED file. This is a tab-separated file with four columns labelled with headings:
  - **Chromosome**
  - **Start** – start position from the analyzed BED file
  - **End** – end position from the analyzed BED file
  - **Custom.Exon** – custom exon name
- Custom reporting – Boolean value indicating whether an output file containing only calls in genes with custom annotation should be generated. The default value is FALSE.
- Output prefix – the prefix for the output files. If none is supplied, the default value is DECoN.
- Plotting of variants – takes one of “All”, “Custom”, or “None”, will create an automated visualization (plot) for either all variants, variants in exons which have custom annotation, or no variants, respectively. The default value is “All”.
- Plot folder – the folder in which plots are saved, created if it doesn’t exist already. Defaults to “DECoNPlots”.

**4.3.2 Running makeCNVcalls**

In Mac/Linux, run the following command from the directory containing the DECoN scripts:

**Rscript makeCNVcalls.R --Rdata *summary.file* --transProb *transition.probability* --exons *customNumbers.file* --custom *FALSE* --out *output.prefix* –-plot *All* --plotFolder *DECoNPlots***

In Windows, click the *makeCNVcalls.bat* executable. You will be prompted to enter inputs.

**4.3.3 Output**

makeCNVcalls outputs two files:

- Summary RData file - a summary .RData file containing the FPKM for each sample and exon, all CNV calls, and quality control information
- Table of all calls – a tab-separated text file ending in *_all.txt* detailing all exon CNV calls. This file has 15 columns:
  - **CNV.ID** – a unique identifier of the CNV call. This ID is searchable in the GUI.
  - **Sample** – the name of the sample
  - **Correlation** – the maximum correlation between the test sample and any other sample in the full set of BAM files
  - **N.comp** – the number of samples used as the reference set
  - **Start.b** – the number of the first exon in the call from the analyzed BED file
  - **End.b** - the number of the last exon in the call from the analyzed BED file
  - **CNV.type** – the type of call. This column has a value of either “deletion” or “duplication”
  - **N.exons** – the number of exons encompassed by the call
  - **Start** - the start position of the call from the analyzed BED file
  - **End** - the end position of the call from the analyzed BED file
  - **Chromosome –** the chromosome number of the call from the analyzed BED file
  - **Genomic.ID** – an identifier of the format **Chromosome:Start-End**
  - **BF** – the Bayes factor associated with the call, generated by DECoN.
  - **Reads.expected** – the number of expected reads under the probabilistic model
  - **Reads.observed** – the number of observed reads
  - **Reads.ratio** – the ratio of observed to expected reads
  - **Gene –** the affected gene from the analyzed BED file
  - **Custom.first** – the number of the first exon in the call from the custom exon numbering file. If the exon is not in the custom exon numbering file NA is given.
  - **Custom.last** – the number of the last exon in the call from the custom exon numbering file. If the exon is not in the custom exon numbering file NA is given.

If a single call spans multiple genes on the same chromosome, the call will be represented with multiple entries (one per gene) in the output. The gene-specific information will be given in the Start.b, End.b, Gene, Custom.first and Custom.last columns. All other columns will have the same values for all entries for the call.

If the Custom option is TRUE and calls are identified which affect a gene with custom annotation, an additional file ending in *_custom.txt* is created. This is the same format as the *_all.txt* file and contains the subset of information pertaining to the relevant genes.

If the plotting option is either “All” or “Custom”, then a simple visualization of each variant is automatically created in the plot folder. The plot shows the variant compared to the reference samples and the ratio of the observed to expected coverage for the sample with the variant. A 95% confidence interval for the ratio of observed to expected coverage is also displayed. All exons in the gene are shown. If the variant is within five exons of the gene endpoints an additional five exons from a neighbouring gene (as specified by the BED file) are also shown. Custom numbering is used if supplied by the user, otherwise exons are numbered in increasing chromosome order within each gene. An example is given in Section 5.4.3.

**4.4 Visualizing calls**

The DECoN call visualization step provides interactive exploration and visualization of the exon deletion/duplication calls and the underlying data.

**4.4.1 Input**

This step has one required input:

- Summary RData file – a summary .RData file containing the FPKM for each sample and exon, all CNV calls, and quality control information, generated in Section 4.3.

**4.4.2 Running DECoN call visualization**

In Mac/Linux, run the following command from the directory containing the DECoN scripts:

**Rscript runShiny.R --Rdata *summary.file***

In Windows, click the *gui.bat* executable. You will be prompted to enter inputs.

**4.4.3 Output**

This step automatically launches a web browser with an interactive GUI. Further details and examples are provided in Section 5.4.4.


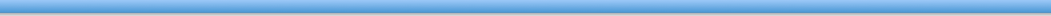


**5 EXAMPLES**

Two examples are presented in this section, describing exon CNV calling and visualization using data generated from a targeted panel. The input dataset is described in Section 5.1. Example 1 describes analysis of the dataset using DECoN in a Mac or Linux environment and is presented in Section 5.2. Example 2 describes analysis of the same dataset in a Windows environment and is presented in Section 5.3. The resulting outputs are the same in all environments and are described in Section 5.4.

**5.1 Input**

The input dataset has 48 samples to be analyzed in a single batch. Each sample has a BAM file and a BAI file. The targeted panel is specified in a sorted 1-based BED file called *Target_Regions.bed*. The reads were aligned to the hg19 reference genome FASTA file called *hg19.fa*. The custom annotation for *BRCA1* and *BRCA2* are specified in a text file called *customNumbering.txt*. The twelve aligned BAM and BAI files, the BED file, the reference genome FASTA file, and the custom annotation text file are all stored in a folder called *test_files*. Examples of the inputs are shown below.

**5.1.1 BAM files**

The BAM files are described in a file called *bamList.txt*. The first five lines are shown below:

test_files/101.bam

test_files/102.bam

test_files/103.bam

test_files/104.bam

test_files/105.bam

DECoN requires that every BAM file have a BAI file in the same folder, named in the same way but with a .bai file extension. For example, sample 101 has the files 101.bam and 101.bai in the *test_files* folder.

**5.1.2 BED file**

A subset of the *Target_Regions.bed* file describing three exons of *BRCA1* is shown below:

17 41258473 41258550 BRCA1

17 41267743 41267796 BRCA1

17 41276034 41276113 BRCA1

The BED file does not have a header. The coordinates are 1-based, i.e. the first targeted base for the first exon above is 41258473 on chromosome 17 of the reference genome file *hg19.fa*.The file is sorted in increasing chromosome order.

**5.1.3 Custom annotation text file**

The header of the *customNumbering.txt* file and a subset describing the three exons of *BRCA1* shown in Section 5.1.2 is shown below:

Chr Start End Gene Custom.Exon

17 41258473 41258550 BRCA1 5

17 41267743 41267796 BRCA1 3

17 41276034 41276113 BRCA1 2

The first four columns are the same for the exons of *BRCA1*. The Custom Exon column shows the use of custom exon numbering for *BRCA1*.

**5.2 Example 1 – Mac/Linux**

In a Mac/Linux environment, the analysis steps are run using the command line. The commands are provided and explained below.

**Rscript ReadInBams.R --bams *bamList.txt* --bed *test_files/Target_Regions.bed* --fasta *test_files/hg19.fa* --out *DECoNtest***

This command reads the BAM files and generates the FPKM for each exon and each sample, outputting a summary .RData file called DECoNtest.RData (Section 4.1).

**Rscript IdentifyFailures.R --Rdata *DECoNtest.RData* --exons *test_files/customNumbering.txt* --custom TRUE --out *DECoNtest***

This command uses the summary DECoNtest.RData file and default thresholds to identify any exons and/or samples where exon CNV calling may be suboptimal (Section 4.2). The custom option is set to TRUE thus two text files will be created if any samples or exons are identified which affect a gene with custom annotation, called DECoNtest_Failures.txt and DECoNtest_custom_Failures.txt. These are described in detail in Section 5.4.1.

**Rscript makeCNVcalls.R --Rdata *DECoNtest.RData* --exons *test_files/customNumbering.txt* --custom TRUE --out *DECoNtestCalls --*plot *All* –-plotFolder *DECoNTestPlots***

This command uses the summary DECoNtest.RData file and default parameters to call exon CNVs (Section 4.3). The custom option is set to TRUE thus two text files are created, called DECoNtest_all.txt and DECoNtest_custom.txt. These are described in detail in Section 5.4.2. Full information is outputted to a summary .RData file called DECoNtestCalls.RData. Plots of all variants are created in the folder DECoNTestPlots.

**Rscript runShiny.R --Rdata *DECoNtestCalls.RData***

This command launches the interactive GUI in a web browser. Visualization examples are described in Section 5.4.3.

**5.3 Example 2 – Windows**

In a Windows environment, the analysis steps are run by double-clicking the .bat file and responding to the interactive prompts in the terminal window. Stdout and stderr are automatically redirected to log files. The four .bat files to run sequentially are:

**ReadInBams.bat**

**IdentifyFailures.bat**

**makeCNVcalls.bat**

**gui.bat**

The resulting .txt files and visualization examples are described in Section 5.4.

**5.4 Outputs**

**5.4.1 IdentifyFailures output**

Below is the full output, DECoNtest_Failures.txt, from the IdentifyFailures step (Section 4.2):

**Sample Exon Type Gene Custom.numbering Info**

Sample_126 All Whole sample All NA Low correlation: 0.977442970434687

All 87 Whole exon EPCAM NA Low median read depth (FPKM): 58

All 347 Whole exon PMS2 NA Low median read depth (FPKM): 79

All 514 Whole exon RECQL4 NA Low median read depth (FPKM): 32.5

All 515 Whole exon RECQL4 NA Low median read depth (FPKM): 41

All 1185 Whole exon NF1 NA Low median read depth (FPKM): 97.5

All 1344 Whole exon STK11 NA Low median read depth (FPKM): 69.5

All 1382 Whole exon SMARCB1 NA Low median read depth (FPKM): 33

One sample, Sample_126, has a highest correlation of 0.977 with the other samples, below the default threshold of 0.98. Seven exons have median coverage across all samples below the minimum default threshold of 100. The number indicating each exon’s order in the Target_Regions.bed file is given in the Exon column. None of these exons were in *BRCA1* or *BRCA2* and thus all seven have NA in the Custom.numbering column.

In this example, there were no failures in genes with custom annotation, thus no DECoNtest_custom_Failures.txt file was created.

**5.4.2 makeCNVcalls output**

Table 1 below contains a subset of the output from the makeCNVcalls step, DECoNtest_all.txt.

| CNV.ID | Sample | Correlation | N.  comp | Start.b | End.b | CNV.Type | N.  exons | Start | End | Chr | Genomic.ID | BF | Reads.  expected | Reads.  observed | Reads.  ratio | Gene | Custom.  first | Custom.last |
| --- | --- | --- | --- | --- | --- | --- | --- | --- | --- | --- | --- | --- | --- | --- | --- | --- | --- | --- |
| 49 | Sample_119 | 0.998648 | 8 | 1257 | 1257 | deletion | 1 | 41209070 | 41209152 | 17 | chr17:41209070-41209152 | 7.99 | 321 | 205 | 0.639 | BRCA1 | 20 | 20 |
| 50 | Sample_120 | 0.996575 | 3 | 315 | 315 | deletion | 1 | 176562106 | 176563031 | 5 | chr5:176562106-176563031 | 16.4 | 673 | 389 | 0.578 | NSD1 | NA | NA |
| 51 | Sample_120 | 0.996575 | 3 | 347 | 349 | duplication | 3 | 6013031 | 6018327 | 7 | chr7:6013031-6018327 | 5.24 | 1389 | 1666 | 1.2 | PMS2 | NA | NA |
| 52 | Sample_120 | 0.996575 | 3 | 1421 | 1428 | deletion | 16 | 14861690 | 133119476 | X | chrX:14861690-133119476 | 111 | 16836 | 11990 | 0.712 | FANCB | NA | NA |
| 52 | Sample_120 | 0.996575 | 3 | 1429 | 1436 | deletion | 16 | 14861690 | 133119476 | X | chrX:14861690-133119476 | 111 | 16836 | 11990 | 0.712 | GPC3 | NA | NA |
| 53 | Sample_121 | 0.988664 | 9 | 1264 | 1269 | deletion | 6 | 41234422 | 41251897 | 17 | chr17:41234422-41251897 | 56.2 | 6145 | 3506 | 0.571 | BRCA1 | 8 | 13 |

Table 1: Subset of DECoNtest_all.txt

| CNV.ID | Sample | Correlation | N.  comp | Start.b | End.b | CNV.Type | N.  exons | Start | End | Chr | Genomic.ID | BF | Reads.  expected | Reads.  observed | Reads.  ratio | Gene | Custom.  first | Custom.last |
| --- | --- | --- | --- | --- | --- | --- | --- | --- | --- | --- | --- | --- | --- | --- | --- | --- | --- | --- |
| 49 | Sample_119 | 0.998648 | 8 | 1257 | 1257 | deletion | 1 | 41209070 | 41209152 | 17 | chr17:41209070-41209152 | 7.99 | 321 | 205 | 0.639 | BRCA1 | 20 | 20 |
| 53 | Sample_121 | 0.988664 | 9 | 1264 | 1269 | deletion | 6 | 41234422 | 41251897 | 17 | chr17:41234422-41251897 | 56.2 | 6145 | 3506 | 0.571 | BRCA1 | 8 | 13 |

Table 2: Subset of DECoNtest_custom.txt

An example of a single exon call is shown in the first call in Table 1, CNV ID 49 made in Sample_119. This sample has a maximum correlation of 0.9986 with other samples in the batch. Eight other samples were used as the reference set. This call affects a single exon, number 1257 in the *Target_Regions.bed* file, which is custom exon 20 in *BRCA1*. It is a deletion called with a Bayes factor of 7.99 based on observing 205 reads instead of the expected 321 reads, a read ratio of 0.639.

An example of a multi-gene call is shown in the fourth call in Table 1, CNV ID 52 made in Sample_120. This call has entries in the fourth and fifth rows for the two affected genes, *FANCB* and *GPC3*. Both rows show that this sample has a maximum correlation of 0.9996575 with other samples in the batch. Three other samples were used as the reference set. This call affects eight exons in *FANCB*, numbers 1421-1428 in the *Target_Regions.bed* file, and eight exons in *GPC3*, numbers 1429-1436 in the *Target_Regions.bed* file. No custom numbering is given for these exons as none was supplied in the custom numbering file.

An example of a multi-exon call is shown in the fifth call in Table 1, CNV ID 53 made in Sample_121. This sample has a maximum correlation of 0.9886 with other samples in the batch. Nine other samples were used as the reference set. This call affects six exons, numbers 1264-1269 in the *Target_Regions.bed* file, which are custom exons 8-13 in *BRCA1*. It is a deletion called with a Bayes factor of 56.2 based on observing 3506 reads instead of the expected 6145 reads, a read ratio of 0.571.

Output was also generated for only genes in the custom numbering file in the file DECoNtest_custom.txt, a subset of which is shown in Table 2 above. Table 2 is an exact subset of Table 1, containing only the calls in *BRCA1* and *BRCA2* as these were the genes present in *customNumbering.txt*.

**5.4.3 Plots**

The automatically generated plot for CNV ID 53 is shown in Figure 1. At the top, the coverage for the test sample – the sample containing the variant – is shown in blue. The coverage for all reference samples are shown in grey. The variant is shown in red. The bottom of the plot shows the ratio of observed to expected coverage for the test sample, along with the 95% confidence interval in grey.


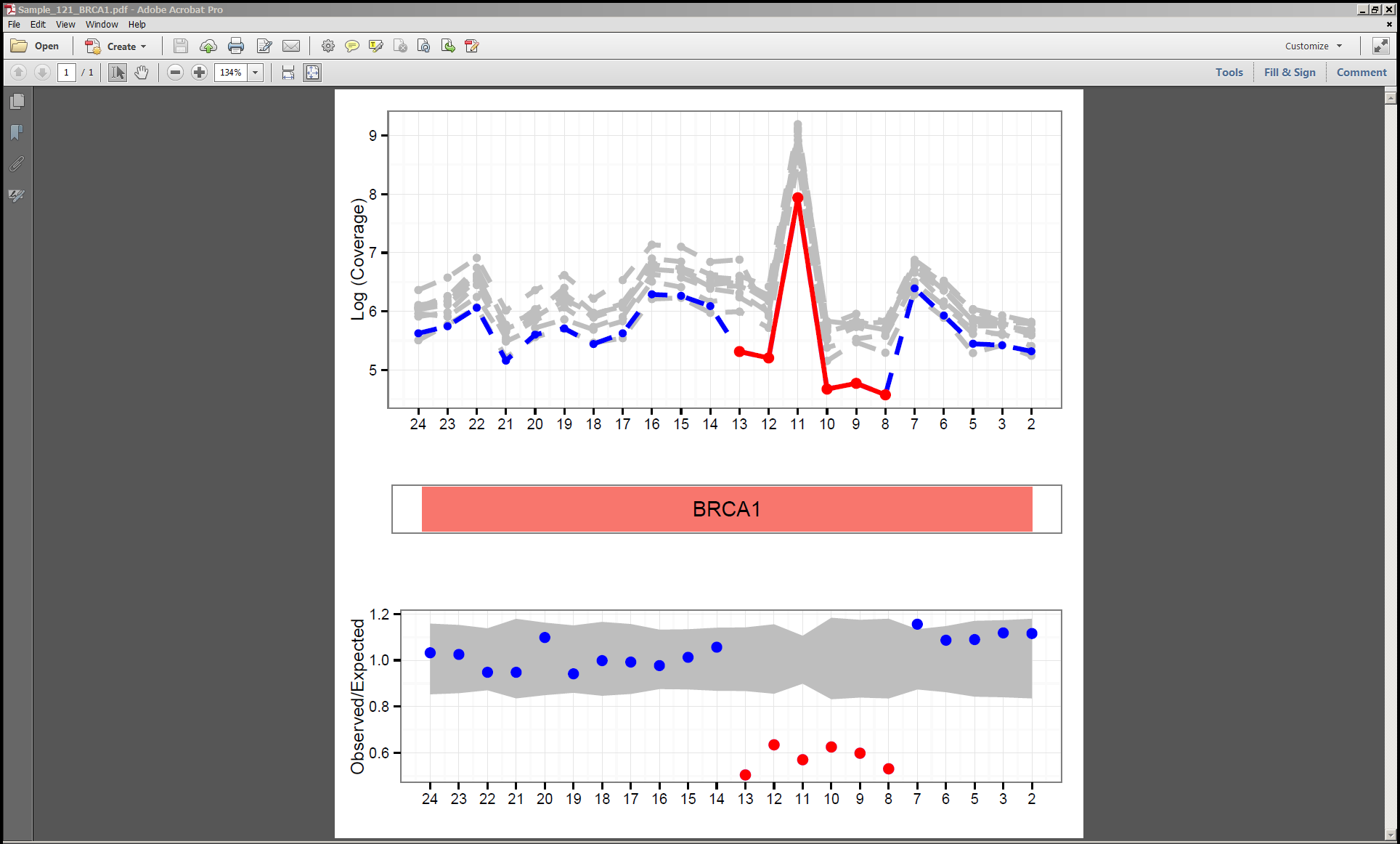


**Figure 1: Plot of a deletion of exons 8-13 in *BRCA1***

**5.4.3 GUI interaction**

The interactive GUI (Section 4.4.3) is presented in a web browser. A number of features are available and presented below.

**Data**

The Data tab allows the user to review the input data (Figure 2). Clicking on the button for the BED file, the BAM files and sample names, or the FASTA file will display the relevant information.

| 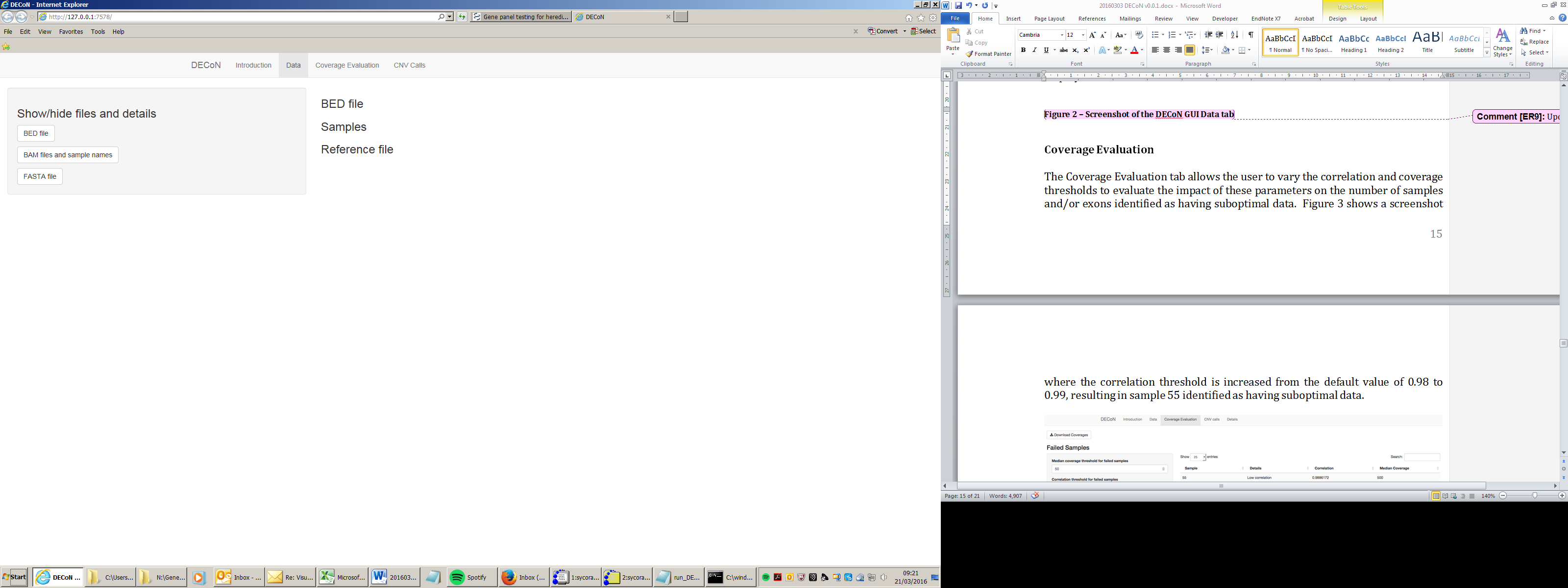 |
| --- |

Figure 2 – Screenshot of the DECoN GUI Data tab

**Coverage Evaluation**

The Coverage Evaluation tab allows the user to vary the correlation and coverage thresholds to evaluate the impact of these parameters on the number of samples and/or exons identified as having suboptimal data. Figure 3 shows a screenshot where the correlation threshold is increased from the default value of 0.98 to 0.99, resulting in five samples identified as having suboptimal data.

| 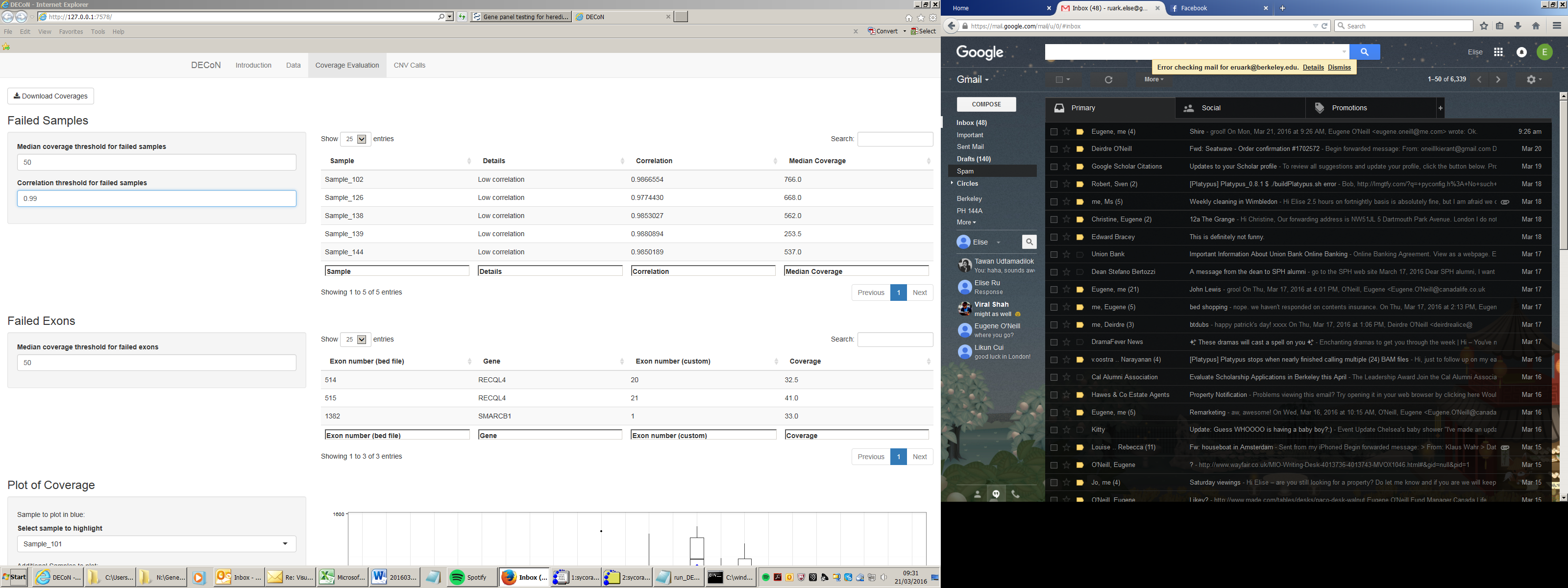 |
| --- |

Figure 3 – Screenshot of the DECoN GUI Coverage Evaluation tab.

The Coverage Evaluation tab also allows plotting of samples or exons with suboptimal data to aid visualization of results. The user can select the samples to be plotted, the scales used in the plot, and the genes to be plotted. Figure 4 shows a screenshot where Sample_101 is plotted in blue with its six reference samples in grey, for genes *BRCA1* and *BRCA2*. Exons are numbered according to the order of the BED file.

| 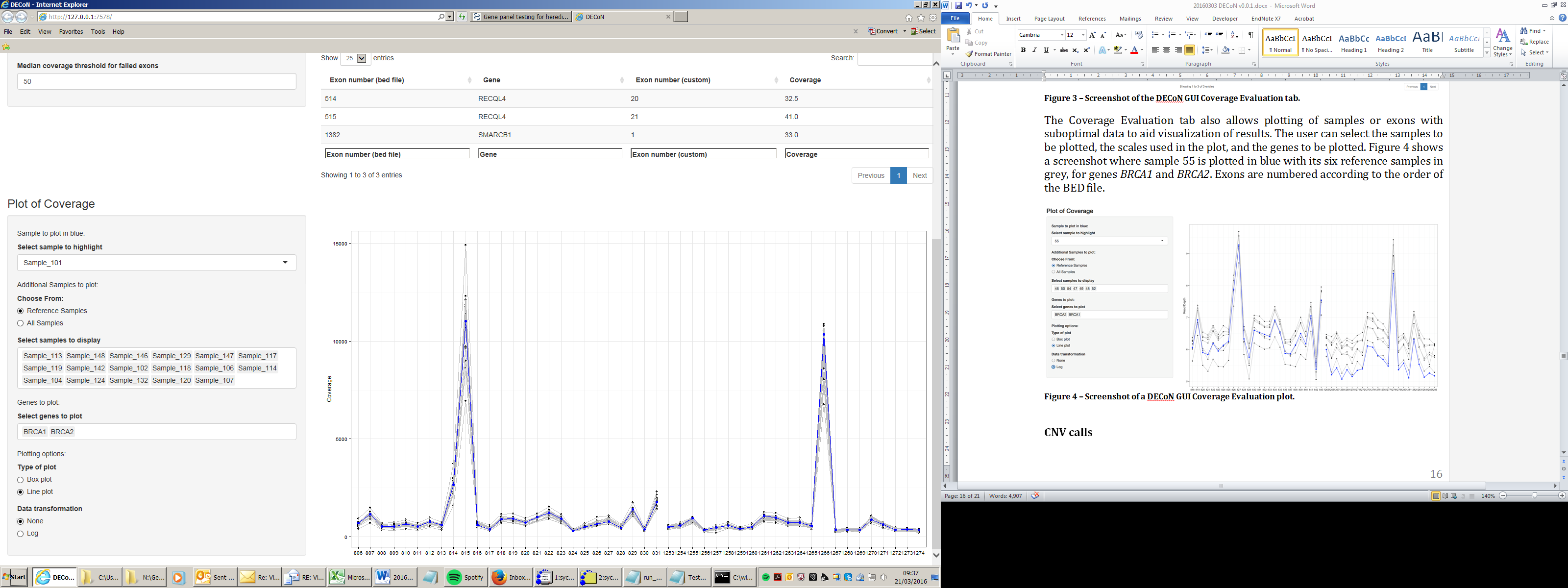 |
| --- |

Figure 4 – Screenshot of a DECoN GUI Coverage Evaluation plot.

**CNV Calls**

The CNV Calls tab shows a table of all exon CNV calls detected by DECoN. The calls can be searched in multiple ways using the different search boxes below the table. Figure 5 shows a screenshot of the table filtered using the gene search “BRCA”. There are 17 calls which fulfill this search criterion with ten shown at a time.


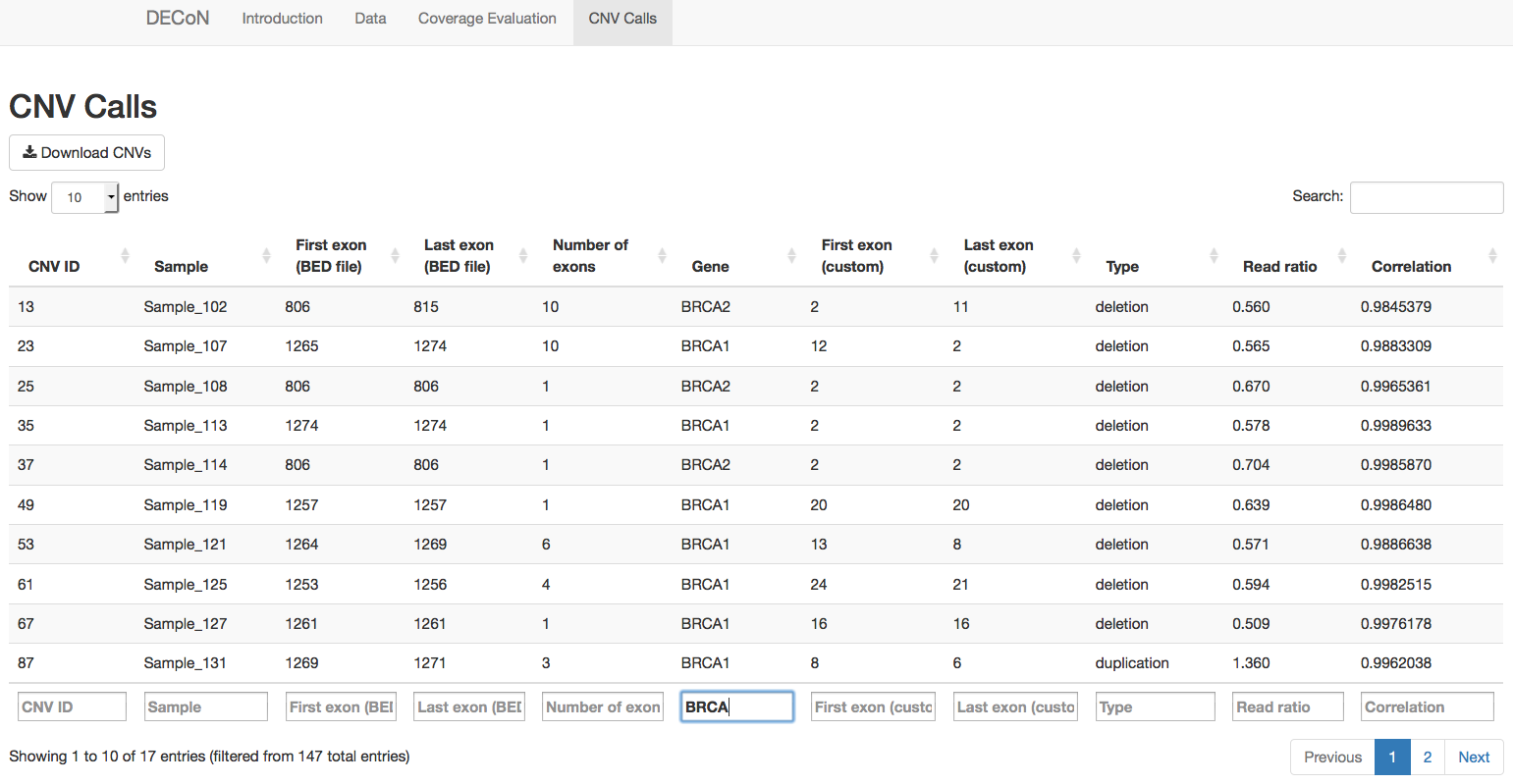


|  |
| --- |

Figure 5 – Screenshot of the DECoN GUI CNV calls table.

This tab also allows plotting of individual calls for further visualization of results. The user can select the CNV ID to be plotted, the number of exons displayed in the plot, the other samples to be plotted (either its reference set or the full set), and the scale of the plot. The affected exon(s) is shown in red and a 95% confidence interval is also shown in grey. Screenshots of CNV IDs 49 and 53 are shown in Figures 6 and 7, respectively.

| 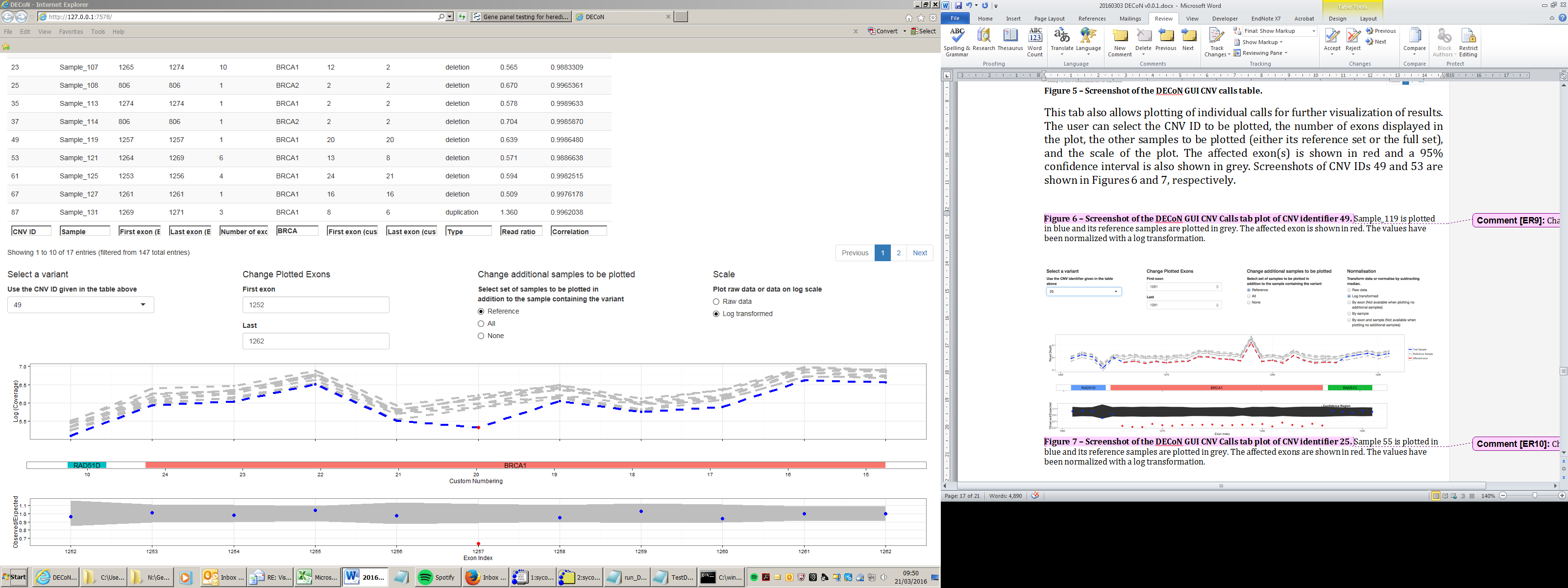 |
| --- |

Figure 6 – Screenshot of the DECoN GUI CNV Calls tab plot of CNV ID 49. Sample_119 is plotted in blue and its reference samples are plotted in grey. The affected exon is shown in red. The values have been normalized with a log transformation.

| 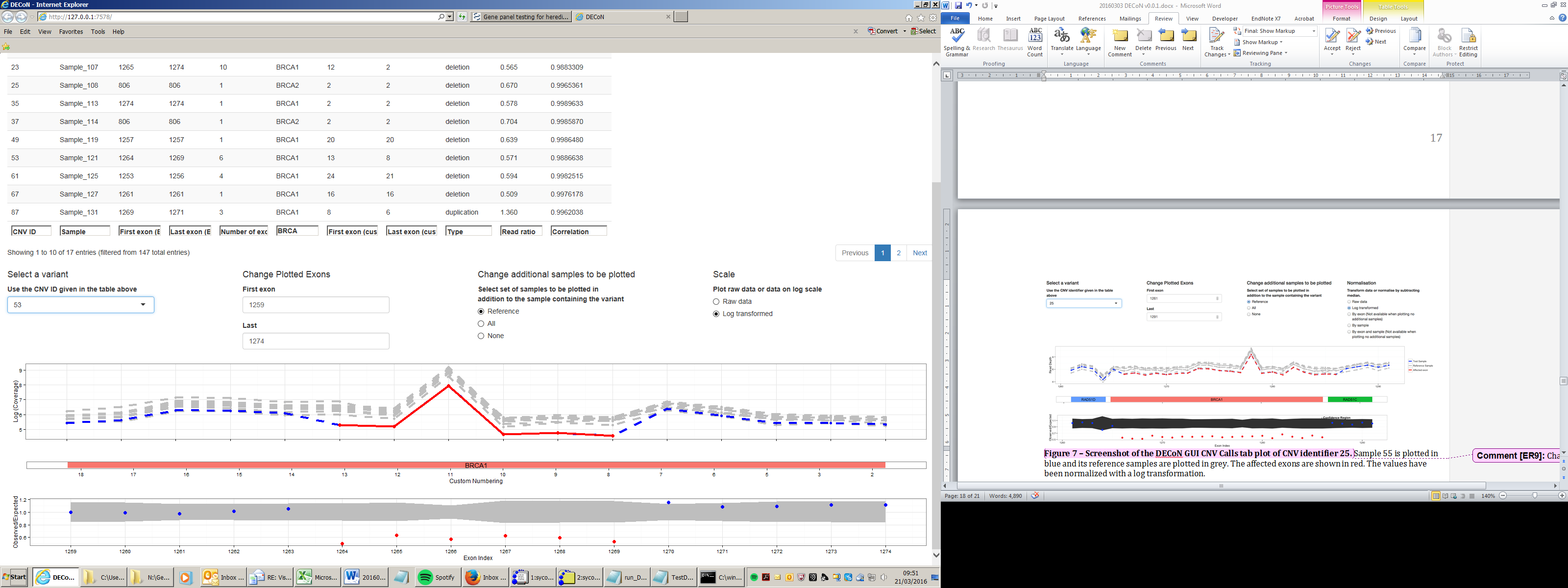 |
| --- |

Figure 7 – Screenshot of the DECoN GUI CNV Calls tab plot of CNV ID 53. Sample_121 is plotted in blue and its reference samples are plotted in grey. The affected exons are shown in red. The values have been normalized with a log transformation.


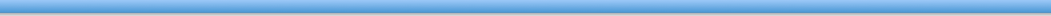


**6 REFERENCES**

1 Plagnol, V. *et al.* A robust model for read count data in exome sequencing experiments and implications for copy number variant calling. *Bioinformatics* **28**, 2747-2754, doi:10.1093/bioinformatics/bts526 (2012).

2 R: A language and environment for statistical computing (Vienna, Austria, 2014).

3 packrat: A Dependency Management System for Projects and their R Package Dependencies v. 0.4.3 (2015).


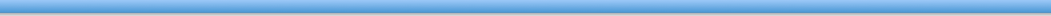


**7 CONTACT**

Please submit all bug reports, comments, questions and feature requests in the DECoN User Group on Google Groups:

<https://groups.google.com/forum/#!forum/decon-user-group>

Feedback can also be sent via email to decon-user-group@googlegroups.com.


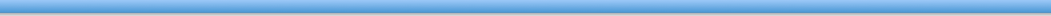


**8 APPENDIX – Rtools download and installation**

This document provides step by step instructions for download and installation of Rtools.

1. Download Rtools32.exe from <http://cran.r-project.org/bin/windows/Rtools/> (highlighted in purple in the screenshot below):


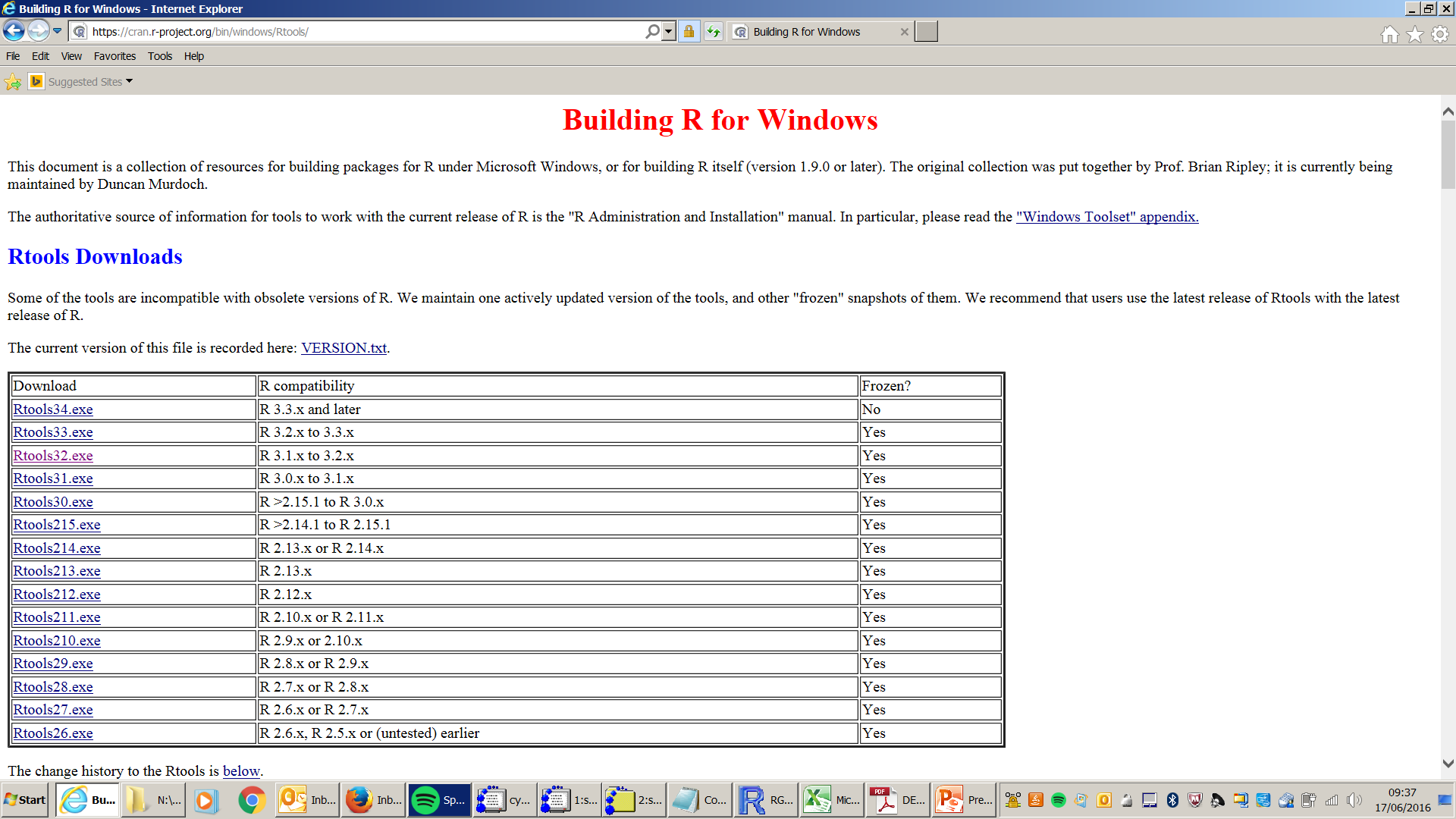


1. Once downloaded, open the .exe file.
2. Select **Run**, then **Yes** to allow changes to be made by the program. Select the language to use during the installation.
3. Once the installation wizard starts, click **Next** to begin, and **Next** to accept the terms and conditions.
4. Click **Next** to accept the default install location. If you choose to change the default location make a note of the new installation path as this will be required later.
5. Click **Next** to accept the default components.
6. Make sure the box is ticked to edit the path variable:


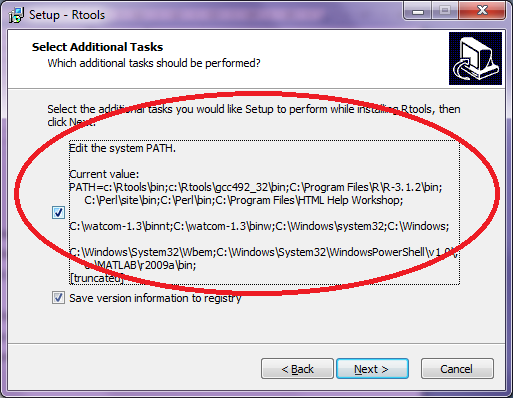


1. Click **Next**.
2. Make sure that the file path to Rtools is in the list; if you altered the install location earlier, this path needs to match: **
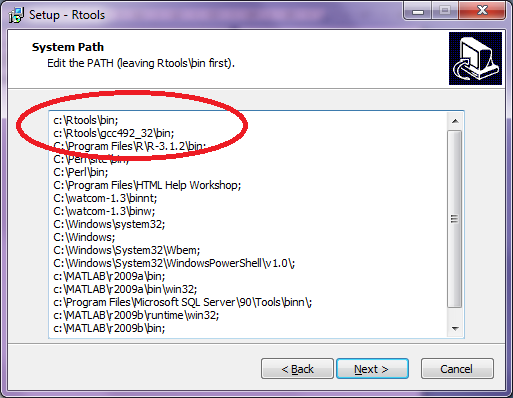
**
3. Click **Next.**
4. Click **Install**.
