# Supplementary material for: Accurate clinical detection of exon copy number variants in a targeted NGS panel using DECoN
Source: Wellcome Open Res. 2016 Nov 25;1:20. [Version 1] doi: 10.12688/wellcomeopenres.10069.1 (PMC5409526; doi:10.12688/wellcomeopenres.10069.1)
Supplement: Supplementary file 3 [file wellcomeopenres-1-10849-s0002.tgz › b2159394-0e53-4b4a-a205-152085097d59.docx]

**Supplementary File 3. Exon CNV detections in the clinical implementation set.**

Exon CNVs in the clinical implementation set detected by DECoN and confirmed by multiplex ligation-dependent probe amplification.

| **Gene** | **Exon CNV** |
| --- | --- |
| BRCA1 | Exon 13 duplication |
| BRCA1 | Exon 13 duplication |
| BRCA1 | Exon 13 duplication |
| BRCA1 | Exon 13 duplication |
| BRCA1 | Exon 13 duplication |
| BRCA1 | Exon 3-8 duplication |
| BRCA1 | Exon 1-2 deletion |
| BRCA1 | Exon 8 deletion |
| BRCA1 | Exon 16 deletion |
| BRCA1 | Exon 20 deletion |
| BRCA1 | Exon 20 deletion |
| BRCA1 | Exon 20 deletion |
| BRCA1 | Exon 20 deletion |
| BRCA1 | Exon 20 deletion |
| BRCA1 | Exon 24 deletion |
| BRCA1 | Exon 7-8 deletion |
| BRCA1 | Exon 21-24 deletion |
| BRCA2 | Exon 1-2 deletion |
| BRCA2 | Exon 3 deletion |
| BRCA2 | Exon 12-13 deletion |
| BRCA2 | Exon 14-16 deletion |
| BRCA2 | Exon 14-16 deletion |
| BRCA2 | Exon 14-16 deletion |
