# Supplementary material for: Accurate clinical detection of exon copy number variants in a targeted NGS panel using DECoN
Source: Wellcome Open Res. 2016 Nov 25;1:20. [Version 1] doi: 10.12688/wellcomeopenres.10069.1 (PMC5409526; doi:10.12688/wellcomeopenres.10069.1)
Supplement: Peer review history

# Peer review: Referee response for version 1

**DOI:** 10.21956/wellcomeopenres.10849.r21828

**Published:** 2017-04-12

**Authors:** Yvonne Wallis, Samuel Clokie

**Affiliations:** 1 West Midlands Regional Genetics Laboratory, Birmingham, UK

This paper uses a tool that appears to address a recognised requirement (assessment of exon CNVs using a panel testing strategy). It will be interesting to see how this approach may be successfully adopted by other centres.

 In general the paper was written clearly however there was a little confusion regarding the sequencing depth routinely used for the analysis and what is recommended when running samples on the MiSeq and HiSeq platforms, including batch sizes. Some further clarification about the algorithm itself may add value to the paper, including whether it uses the same approach as ExomeDepth of removing outliers from a batch, whether a training set is required and if so how many.

We have read this submission. We believe that we have an appropriate level of expertise to confirm that it is of an acceptable scientific standard.

# Peer review: Referee response for version 1

**DOI:** 10.21956/wellcomeopenres.10849.r20638

**Published:** 2017-03-01

**Authors:** Birgit Sikkema-Raddatz, Lennart F. Johansson

**Affiliations:** 1 Department of Genetics,University Medical Centre Groningen, University of Groningen, Groningen, Netherlands; 2 Faculty of Medical Sciences, University of Groningen, Groningen, Netherlands

The manuscript is clearly written and DECoN looks like a promising tool for a difficult to tackle problem. In general the article gives a good overview of the strength and performance of the tool. However, the article would benefit if some extra information, as described in the points below, was added. As a personal interest I would like to hear more about the quality metrics used to flag low quality samples and exons. This really looks like one of the strong points of the method.

**Minor points:**

***P3*. **There are other tools published that also specifically target exon copy number variants in targeted gene panels, such as CoNVaDING.

***P3. ***The TSCP contains 94 genes. An explanation is needed why only 10 of those genes are used as evaluation set.

***P4. ***‘DECoN relies on a high level of correlation between samples’. It is unclear if this correlation should already be present in the samples analyzed (by analyzing only samples belonging to the same pool) or if DECoN performs a correlation calculation and based on that calculation selects a subset of samples for further calculations. Can DECoN also analyze samples that are not sequenced in the same pool?

***P4. ***Is the correlation score in the report used as a quality metric, and if yes, how? And how were the correlation scores for the samples analyzed?

***P4. ***What are the suggested thresholds to flag samples /exons which may have suboptimal performance? Were such thresholds used in the evaluation set? Is the Bayes factor used as part of the quality metric? And if yes, ***P6.*** how many samples and exons were flagged? In my opinion this is a critical step in analysis. For clinical detection it is just as important to know which exons can’t be reliably analyzed in the data as to know which can. This creates the difference between a false negative result and a failed sample/failed exon analysis. What was the flag status of the exon 8-11 FP duplication.

***P6. ***Why were simulated samples entirely comprised of samples with a exon CNVs excluded from specificity calculations. There can still be a false positive result in other (two copy) exons.

***P6. ***Please state explicitly the specificity of the 8 non-BRCA genes in the evaluation set, even though this is 100%.

***P6. ***It would also be interesting to know the specificity and sensitivity for the other 84 genes in the TSCP.

***P6. ***How is the quality of the evaluated exons compared to the other exons in the TSCP? Can the sensitivity and specificity and exons passing quality control be extrapolated to genes outside the evaluation panel?

***P6 and table 2.*  **Instead of the number of reads per sample I would mention the average coverage / read depth of the evaluated exons. This is a more informative value, since it is independent of panel size.

***P7, figure 2: *** What is the flag status of exon 20 in the duplication set? In other words, why does this exon perform worse than the others, especially since the sensitivity for deletions is so high

***P7, figure 2: *** Can dots for specificity be added to the graph?

***P8. ***How does DECoN perform compared to the other named tools?

***P8. ***Is DECoN tailor made for these 10 TCSP genes analyzed in pools, or is the performance generalizable to other targeted capturing panels?

We have read this submission. We believe that we have an appropriate level of expertise to confirm that it is of an acceptable scientific standard.
